# The Impact of Endoscopic Dilatation in Post-Esophagectomy Anastomotic Stricture: Risk Factors and Treatment Outcomes

**DOI:** 10.7759/cureus.104013

**Published:** 2026-02-21

**Authors:** Murad Jamal, Muhammad Mudasir, Shehzad Khan, Hala Mansoor, Kiran Jalil, Muhammad Nauman Shahid, Sabeen Farhan

**Affiliations:** 1 Internal Medicine, Shaukat Khanum Memorial Cancer Hospital and Research Centre, Lahore, PAK; 2 Gastroenterology, Shaukat Khanum Memorial Cancer Hospital and Research Centre, Lahore, PAK; 3 Gastroenterology and Hepatology, Shaukat Khanum Memorial Cancer Hospital and Research Centre, Lahore, PAK; 4 Gatsroentrology, Shaukat Khanum Memorial Cancer Hospital and Research Centre, Lahore, PAK; 5 Gastroentrology, Shaukat Khanum Memorial Cancer Hospital and Research Centre, Lahore, PAK; 6 Orthopedic Surgery, Ghurki Trust Teaching Hospital, Lahore, PAK

**Keywords:** anastomotic stricture, dysphagia, endoscopic dilatation, esophageal stent, esophagectomy, refractory stricture

## Abstract

Background: Benign anastomotic strictures are a common complication after esophagectomy, contributing significantly to postoperative morbidity and reduced quality of life. Endoscopic dilatation is the primary treatment modality; however, a subset of patients develop refractory strictures that require repeated interventions and pose unique therapeutic challenges.

Objectives: To evaluate the clinical impact of endoscopic dilatation in patients with post-esophagectomy strictures and to identify procedural and clinical indicators associated with stricture refractoriness.

Methods: This retrospective observational study analyzed 69 patients who developed benign anastomotic strictures after esophagectomy and underwent endoscopic dilatation at Shaukat Khanum Memorial Cancer Hospital and Research Centre (SKMCH&RC), Lahore, Pakistan between January 2019 and December 2023. Patients were categorized as having recurrent (n = 61) or refractory strictures (n = 8) based on response to treatment. Demographics, procedural variables, and outcomes were assessed using Statistical Package for the Social Sciences (SPSS) version 25, with statistical significance set at *p* < 0.05.

Results: Refractory strictures accounted for 7(11.6%) of cases and were associated with a significantly higher number of dilatation sessions (mean 12.25 vs. 4.70; *p* < 0.001), smaller maximum dilator sizes achieved (12.63 mm vs. 13.77 mm; *p* = 0.002), and earlier onset of symptoms requiring intervention (3.5 vs. 5.46 months post-surgery; *p* = 0.033). Clinical success after the first session was significantly lower in refractory cases 53 (87.5%) vs. 100 (100%); *p* = 0.005), and stent placement was markedly more frequent 53(87.5%) vs. 15 (24.6%); *p* < 0.001). No statistically significant associations were found between stricture type and demographic variables, tumor staging, or surgical approach.

Conclusion: Endoscopic dilatation is highly effective for managing recurrent post-esophagectomy strictures. However, refractory strictures represent a more aggressive phenotype, characterized by earlier onset, a poor response to initial therapy, and a greater procedural burden. Early identification through procedural indicators, combined with tailored interventional strategies including early stent placement and adjunctive therapies is essential for improving outcomes in this challenging patient subgroup.

## Introduction

Esophageal cancer remains a significant global health challenge, ranking as the eighth most common malignancy and the sixth leading cause of cancer-related mortality worldwide. According to global cancer statistics, more than 570,000 new cases of esophageal cancer are diagnosed annually, with a disproportionately high burden observed in East Asia and parts of Africa [1]. There are two main histological types of esophageal cancer, i.e., squamous cell carcinoma (SCC), which is more prevalent in Asia and Africa, and adenocarcinoma, more commonly seen in Western countries. The 2019 Global Burden of Disease study reported that Pakistan had the highest incidence and prevalence rates among low sociodemographic index Asian countries [2]. Despite advancements in oncological therapies, esophagectomy continues to be the cornerstone of curative intent treatment for resectable esophageal cancers. However, this surgical procedure is associated with a high incidence of postoperative complications, among which anastomotic stricture formation is one of the most common and clinically significant sequelae [3].

Anastomotic strictures occur in 9-40% of patients after esophagectomy. They develop within the first few months and present with progressive dysphagia, odynophagia, regurgitation, or aspiration [4]. The impact on patient quality of life is profound, often leading to recurrent hospital visits, impaired oral intake, weight loss, and increased risk of aspiration pneumonia. Prompt recognition and management of these strictures are essential to ensure optimal postoperative recovery and long-term functional outcomes [5].

Endoscopic dilatation is widely recognized as the first-line, minimally invasive treatment strategy for benign esophageal strictures. Techniques such as Savary-Gilliard bougie dilatation and controlled radial expansion (CRE) balloon dilatation have revolutionized the management of these complications [6]. These approaches offer safe and effective luminal restoration in most cases. Nonetheless, certain subtypes of strictures particularly those that are dense, fibrotic, or ischemic in nature exhibit poor response to standard dilatation protocols. These are classified as refractory and recurrent strictures. These cases often necessitate multiple sessions, temporary stent placement, or even revisional surgery [7].

Multiple factors have been postulated to contribute to the development and recurrence of esophageal strictures, including surgical technique, anastomotic leak, ischemia at the anastomotic site, prior chemoradiotherapy, tumor location and size, histopathological features, and patient-related variables such as diabetes mellitus, obesity, and smoking [8]. Despite a growing body of literature, there remains a lack of consensus regarding the precise predictors of poor response to dilatation and the optimal management algorithms for refractory strictures. Moreover, the role of newer interventions such as biodegradable stents, steroid injection therapy, and adjunctive endoscopic treatments remains underexplored, especially in resource-constrained settings [9].

The present study aims to investigate the impact of endoscopic dilatation on post-esophagectomy stricture resolution, compare treatment outcomes between recurrent and refractory strictures, and identify key demographic, clinical, and procedural risk factors associated with poor response. By characterizing the patterns of stricture behavior and procedural efficacy, this study aspires to enhance our understanding of this complex postoperative complication and contribute to evidence-based, individualized management strategies that improve both functional outcomes and quality of life in esophageal cancer survivors.

## Materials and methods

Study design and setting

This study was conducted as a retrospective cross-sectional study that was carried out in Shaukat Khanum Memorial Cancer Hospital and Research Centre (SKMCH&RC), a tertiary care oncology institution based in Lahore, Pakistan. SKMCH&RC is a national referral center of gastrointestinal malignancies and provides advanced oncological, surgical and endoscopic services.

Study duration

The collected data were taken during the period between January 1, 2019, and December 31, 2023. The study period was deliberately selected to ensure an adequate duration of follow-up, thereby allowing for the inclusion of patients who had undergone multiple endoscopic dilatation procedures. This design facilitated the comprehensive evaluation of both immediate and long-term clinical outcomes.

Study population

The study included adult patients who underwent esophagectomy for histologically confirmed esophageal carcinoma and subsequently developed benign anastomotic strictures. Eligible patients were required to have received at least one session of endoscopic dilatation for stricture management. Based on their response to therapy, patients were classified into recurrent or refractory groups using predefined operational criteria.

Operational definitions

Refractory Stricture

Failure to achieve a luminal diameter of ≥14 mm after at least five consecutive endoscopic dilation sessions performed at two-week intervals.

Recurrent Stricture

Inability to maintain a luminal diameter of ≥14 mm for four weeks after successful dilation (i.e., after achieving ≥14 mm with symptomatic relief), resulting in recurrence of dysphagia and luminal re-narrowing to <14 mm.

Exclusion criteria

The patients were excluded when the etiology of their stricture was not the post-esophagectomy anastomosis, including caustic ingestion, gastroesophageal reflux disease (GERD), or primary radiotherapy but no surgery. Moreover, patients with incomplete records, undocumented operative histories, or insufficient follow-up data were excluded to maintain data integrity and ensure statistical reliability.

Sampling method

A non-probability (census) sampling procedure, one that followed each other in a non-probability manner, was used. The participants were identified using the electronic Health Information System (HIS) of the hospital of the study through the identification of all adult patients who underwent esophagectomy in the period from January 2019 to December 2023, acquired benign anastomotic strictures, and needed endoscopic dilation.

Sample-Size Justification

Since this study is retrospective, descriptive, and is conducted to cover all cases that fit the criteria during the five-year duration, an official calculation of the sample size was not conducted. Every patient who passed through inclusion criteria throughout the study was enlisted to enhance as much representativeness and subgroup comparison as possible.

Data collection procedure

Clinical and procedural data were extracted from the patient charts and electronic medical records using a structured proforma. Variables collected included patient demographics (age, gender), comorbidities (diabetes, hypertension, ischemic heart disease), oncologic data (histologic subtype; Tumor, Node, Metastasis (TNM) classification), surgical details (type of esophagectomy, lymph node dissection), and procedural parameters related to endoscopic dilatation. These procedural variables included the total number of dilatation sessions, the time to first dilatation after surgery, maximum dilator size achieved, use of esophageal stents, and immediate clinical success. All endoscopic interventions were performed by board-certified gastroenterologists, using either Savary-Gilliard bougie dilators or fluoroscopically guided balloon dilators, in accordance with institutional protocols. All medical records were fully de-identified before data extraction, in accordance with institutional policy and Institutional Review Board (IRB) requirements.

Outcome measures

The primary outcomes assessed in the study included the number of dilatation sessions required to achieve symptomatic relief, the maximum luminal diameter attained, the interval between surgeries and the first dilatation and clinical success following the first endoscopic intervention. Clinical success was defined as the resolution of dysphagia and maintenance of adequate swallowing without recurrence within four weeks of the procedure. Secondary outcomes evaluated included associations between stricture behavior (recurrent vs refractory) and patient characteristics such as comorbidities, tumor staging, surgical approach, and stent utilization.

Statistical analysis

All statistical analyses were carried out using IBM Statistical Package for the Social Sciences (SPSS)version 25. Descriptive statistics were employed to summarize continuous variables as means with standard deviations, and categorical variables as frequencies and percentages. The Shapiro-Wilk test was used to assess the normality of data distribution. For between-group comparisons, independent t-tests or Mann-Whitney U tests were applied for continuous variables depending on data normality. Chi-square tests or Fisher’s exact tests were used to analyze categorical variables. Where applicable, effect sizes including Cohen’s d, Hedges’ g, and Glass’s delta were computed to quantify the magnitude of group differences. A two-tailed p-value of less than 0.05 was considered statistically significant throughout the analysis.

## Results

The present study included 69 patients who developed anastomotic strictures following esophagectomy. Based on clinical characteristics and treatment responsiveness, two distinct subgroups were identified: recurrent strictures (n = 61, 54(88.4%)) and refractory strictures (n = 8, 7(11.6%)). The results are organized under thematic subheadings that cover demographic parameters, tumor pathology, surgical details, and endoscopic outcomes. Each section is supported by statistical tables that are clearly cited within the narrative for comprehensive interpretation.

Demographic and clinical profile

No statistically significant demographic and comorbidity differences were found between recurrent and refractory strictures at the baseline levels. The number of male gender cases predominated in the refractory cases (62.5% vs 44.3%, p=0.474), whereas diabetes mellitus, hypertension, and ischemic heart disease exhibited similar low prevalence rates. The history of smoking had a non-significant tendency of greater representation in refractory strictures (25.0% vs 18.0%, p=0.646). The overall p-values, especially those where comorbidities are zero cases on the refractory group are of a statistically low power, not because the groups are necessarily equivalent.

Recurrent and refractory post-esophagectomy strictures do not significantly differ in this cohort of patients based on demographic factor and traditional comorbidities, indicating that stricture behavior is more likely to be dictated by surgical, anatomical, and tumor-associated issues than by patient characteristics or conventional cardiovascular risk factors.

Table 1 presents the detailed comparison of demographic and clinical characteristics between the two groups.

**Table 1:** Demographics and comorbidities by stricture type

Variable	Recurrent (n=61)	Refractory (n=8)	p-value
Male gender, n (%)	27 (44.3%)	5 (62.5%)	0.474
Diabetes mellitus, n (%)	5 (8.2%)	0 (0%)	1.000
Hypertension, n (%)	6 (9.8%)	1 (12.5%)	1.000
Ischemic heart disease, n (%)	2 (3.3%)	0 (0%)	1.000
Smoking, n (%)	11 (18.0%)	2 (25.0%)	0.646

Tumor staging and stricture type

The distribution of the stages of TNM is significantly different at recurrent and refractory anastomotic strictures (p=0.042). Refractory strictures are typified by a far more significant percentage of advanced disease (T3N2/T4: 50.0% vs 14.8%), and recurrent strictures are typified by T3N1 disease (39.3% vs 12.5). It is important to note that no T1-2 early stage tumors were found in the refractory group, so it is possible that significant tumor biology is a risk factor in more recalcitrant stricture development. Refractory strictures are closely linked to advanced TNM stage, whereas the case of T3N1 is dominant in recurrent ones. This difference implies the presence of different pathophysiological processes depending on the original aggressiveness of the tumour. The comparative TNM stage distribution is detailed in Table 2.

**Table 2:** TNM stage distribution by stricture type TNM: Tumor, Node, Metastasis

TNM Category	Recurrent (n=61)	Refractory (n=8)	p-value
Early (T1-2)	7 (11.5%)	0 (0%)	0.545
T3N0	21 (34.4%)	3 (37.5%)	1.000
T3N1	24 (39.3%)	1 (12.5%)	0.083
Advanced (T3N2/T4)	9 (14.8%)	4 (50.0%)	0.021
Overall distribution	-	-	0.042

Surgical approach and lymphadenectomy

There is a specific pattern in the nature of stricture in surgery. The correlation of refractory strictures with transhiatal esophagectomy (50% vs 21%) and few anastomotic foreign bodies (13 vs 54, p=0.037) is observed to have significant correlation, with no difference in anastomotic leak rates, surgical time or pathologic findings. The actions of the surgical approach and anastomotic features have a differentiating impact on stricture behavior, showing transhiatal technique to cause refractoriness, and result in foreign body presence to encourage a recurrent, dilation responsive stricture. The complete comparison of surgical characteristics is provided in Table 3.

**Table 3:** Surgical characteristics by stricture type * Distance measured endoscopically from the upper incisors

Characteristic	Recurrent (n=61)	Refractory (n=8)	p-value
Surgical approach			
Transhiatal esophagectomy, n (%)	13 (21.3%)	4 (50.0%)	0.095
Three-stage esophagectomy, n (%)	44 (72.1%)	4 (50.0%)	0.246
Ivor Lewis/minimally invasive, n (%)	4 (6.6%)	0 (0%)	1.000
Surgical outcomes			
Anastomotic leak, n (%)	3 (4.9%)	0 (0%)	1.000
Duration of surgery, mean ± SD (hours)	5.1 ± 1.3	4.6 ± 0.9	0.293
Pathological findings			
Lymph nodes removed, mean ± SD	1.8 ± 0.6	1.9 ± 0.6	0.615
Histopathological Grade 1, n (%)	25 (41.0%)	3 (37.5%)	1.000
Histopathological Grade 2, n (%)	36 (59.0%)	5 (62.5%)	1.000
Anastomotic details			
Stricture distance from incisors, mean ± SD (cm)*	1.4 ± 0.7	1.8 ± 0.9	0.186
Anastomotic foreign body, n (%)	33 (54.1%)	1 (12.5%)	0.037

Endoscopic and procedural outcomes

There is a similar initial practical success (>87) but different longitudinal courses in endoscopic results. The refractory strictures need a lot more dilatation (9.5 vs 5.7, p=0.010) and fewer treatment periods (1.6 vs 2.8 months, p=0.038), and more adjunctive therapies are needed. Although both groups have similar levels of immediate dysphagia relief, in refractory cases, there is procedural dependency where 88% of refractory cases require more than five dilatations, compared to 44% of recurrent cases (p=0.037). Refractory strictures are a specific clinical presentation that is more difficult to manage in procedures and faster to recur even though the initial outcome of the endoscopy is similar, which requires the move to adjunctive therapies and more intensive follow-up methods.The comprehensive comparison of endoscopic outcomes is provided in Table 4.

**Table 4:** Endoscopic dilatation outcomes by stricture type * Defined as (e.g., an improvement of ≥1 point in the dysphagia score using Mellow-Pinkas score); † Average time (in months) between successive dilatation procedures

Outcome Measure	Recurrent (n=61)	Refractory (n=8)	p-value
Procedural characteristics			
Number of dilatations, mean ± SD	5.7 ± 3.3	9.5 ± 4.5	0.010
Time to first dilation, mean ± SD (months)	4.8 ± 5.2	3.6 ± 2.4	0.514
Maximum dilator size >12mm, n (%)	11 (18.0%)	3 (37.5%)	0.196
Immediate outcomes			
Initial clinical success, n (%)	58 (95.1%)	7 (87.5%)	0.415
Good dysphagia relief, n (%)*	56 (91.8%)	7 (87.5%)	0.521
Adjunctive therapies			
Incision/steroid injection, n (%)	28 (45.9%)	6 (75.0%)	0.145
Stent utilization, n (%)	24 (39.3%)	4 (50.0%)	0.710
Procedural burden			
Patients requiring >3 dilatations, n (%)	41 (67.2%)	8 (100%)	0.093
Patients requiring >5 dilatations, n (%)	27 (44.3%)	7 (87.5%)	0.037
Mean dilatation interval (months)†	2.8 ± 1.9	1.6 ± 0.8	0.038
Complications			
Overall complication rate, n (%)	6 (9.8%)	1 (12.5%)	0.575
Major complications, n (%)	1 (1.6%)	0 (0%)	1.000

Interpretation and clinical implications

The paper has outlined different clinical and pathological characteristics between recurrent and refractory anastomotic strictures after esophagectomy, with significant implications on clinical outcomes and future studies. All of the findings are indicative that these entities are distinct pathophysiological processes, not a spectrum of disease severity. The strong predictive value of advanced TNM stage (T3N2/T4) as a predictor of refractory strictures (odds ratio (OR)=5.78, p=0.021) is a valuable risk stratification variable that can be used to offer specific counseling and increased endoscopic monitoring to high-risk patients, and the relationship between anastomotic foreign bodies and strictures that are responsive to dilation contributes to a more mechanically etiologic group of strictures, which can be more easily managed with conventional treatments. The tendency of more refractory strictures after transhiatal esophagectomy (50% vs 21%, p=0.095) should be taken into account with special attention to the selection of the surgical approach since cervical anastomoses can be preconditioned to various healing biology and undergo technical changes in the optimization of anastomotic perfusion. The significant difference in procedural burden, in which refractory strictures needed 66% more dilatations (9.5 vs 5.7, p=0.010) at shorter intervals (1.6 vs 2.8 months, p=0.038), is supportive of the stratified management protocols in which recurrent strictures could take standard intervals, with refractory cases proceeding to adjunctive therapies at a younger age (incision/steroid injection, etc). This association of refractory strictures with high tumor biology, surgical strategies, and lack of mechanical determinants points to the etiology of the disease being multifactorial in nature resulting from fundamental abnormalities in healing mechanisms combined with surgical concentrations to generate a particular clinical outcome. The significantly greater procedural load in refractory cases (88% of cases requiring more than five dilatations and 44% of recurrent cases, p=0.037) has relevant implications to the allocation of healthcare resources, which justify more intensive but possibly less costly early interventions. The results allow more precise counseling of the patient as per the treatment courses, which facilitates shared decision-making and realistic expectations. An effective clinical guideline is obtained: Patients with high TNM stage and transhiatal esophagectomy must be at risk of refractory strictures; early refractory response that leads to incision/steroid therapy is to be contemplated; the presence of anastomotic foreign bodies should forecast a more responsive dilation course; treatment intervals must be customized according to initial response patterns.

This information, in combination with earlier research, contributes to the use of precision medicine in treating stricture after these cancer surgeries, going beyond standardized regimens to risk-responsive interventions that maximize patient outcomes with minimal procedural costs.

## Discussion

Post-esophagectomy anastomotic strictures management is an issue of concern since it is a continuum of the disease behavior. Our paper defines recurrent and refractory strictures as two distinct pathophysiological entities that have different implications to manage. The high correlation of the advanced tumor biology (T3N2/T4) with the refractory strictures (OR=5.78, p=0.021) indicates the presence of a pro-fibrotic microenvironment, which is retained after surgery, affecting the anastomotic healing [10]. Its association with transhiatal esophagectomy (50% vs 21%, p=0.095) demonstrates that cervical anastomoses, having weaker blood supply and higher mechanical load, can be a risk factor to poor healing [11]. On the other hand, anastomotic foreign bodies caused recurrent dilation responsive strictures (54-13, p=0.037), which suggested a more superficial mechanical etiology [12]. The high procedural cost of refractory cases (66% more dilatations (9.5 vs 5.7, p=0.010)) with almost half of the interval demonstrates their high clinical and financial consequences [13]. The results indicate that there is a multifactorial two-hit model that baseline tumor factors interact with surgical approach to yield different phenotypes [14]. We have a small refractory cohort (n=8) and retrospective design, which requires prospective validation. Clinical implication would involve the creation of risk-stratified algorithms on high-risk patients and the thoughts behind new interventions on abnormal healing biology [15].

Strengths and limitations

A key strength of this study lies in its targeted comparison between recurrent and refractory strictures using well-defined clinical criteria and data from a high-volume cancer treatment center. The dataset provides granular insights into procedural patterns, timing, and therapeutic response. However, several limitations must be acknowledged. The retrospective design inherently introduces selection and documentation bias. The relatively small number of refractory cases (n=8) limits the generalizability of subgroup comparisons and the statistical power for multivariate analysis. Furthermore, although procedural consistency was maintained, the selection of dilator type and the timing of stent placement were determined by the individual endoscopists and not standardized by protocol. Additionally, long-term follow-up data on symptom recurrence, quality of life, and nutritional status were not captured.

Future directions

Prospective studies are required to validate procedural markers of refractoriness and to explore molecular or imaging biomarkers predictive of poor response. The integration of patient-reported outcome measures (PROMs) will be essential for evaluating not just technical success but also functional recovery. Novel endoscopic strategies such as drug-eluting stents or combination therapy with anti-fibrotic agents also warrant investigation. Ultimately, risk stratification tools incorporating clinical, procedural, and possibly biomolecular data could optimize treatment pathways and reduce the healthcare burden associated with refractory post-esophagectomy strictures.

## Conclusions

This paper confirms that recurrent and refractory post-esophagectomy anastomotic strictures are different clinical entities that have divergent risk profiles, treatment responses, and longitudinal courses. The stage of TNM (T3N2/T4) can be recognized as a strong analyst of refractoriness, and a more positive, dilation-responsive recurrence phenotype is associated with the occurrence of an anastomotic foreign body. Refractory strictures have a much higher procedural burden since they require a much higher number of dilations with reduced intervals between. These results confirm a clinically practical classification system that goes beyond a homogenous perspective of anastomotic strictures. They advocate the introduction of risk-stratified algorithms of management, in which a high-risk group of patients (i.e., defined with regards to advanced tumor biology and definite approaches to surgery) may be provided with more stringent surveillance and prompt escalation to adjunctive therapies. The objectives of this precision medicine strategy include maximizing the functional outcomes in the long term, better resource utilization, and patient counseling, thus influencing more effective and personalized treatment courses of esophageal cancer survivors.
